# A transcriptome analysis of the Aedes aegypti vitellogenic fat body

**DOI:** 10.1673/1536-2442(2006)6[1:ATAOTA]2.0.CO;2

**Published:** 2006-06-08

**Authors:** Fabiana M. Feitosa, Eric Calvo, Emilio F. Merino, Alan M. Durham, Anthony A. James, Antonio G. de Bianchi, Osvaldo Marinotti, Margareth L. Capurro

**Affiliations:** 1 Department of Parasitology, Institute of Biomedical Sciences, Universidade de São Paulo, Av. Prof. Lineu Prestes, 1374, Butantan, São Paulo, SP, 05508-000, Brazil; 2 Laboratory of Malaria and Vector Research. National Institutes Health (NIH/NIAID). Rockville, MD 20852, USA; 3 Department of Computer Science, Institute of Mathematics and Statistics, Universidade de São Paulo, 05508-000, SP, Brazil; 4 Department of Molecular Biology and Biochemistry, 2305 McGaugh Hall, University of California, Irvine, CA 92697–3900, USA; 5 Department of Microbiology and Molecular Genetics, University of California Irvine, CA 92697–3900, USA

## Abstract

Aedes (Stegomyia) aegypti is an important dengue vector in tropical and subtropical zones throughout the world. A transcriptome of Ae. aegypti vitellogenic fat bodies is described here. The fat body is a dynamic tissue that participates in multiple biochemical functions of intermediate metabolism. A total of 589 randomly selected cDNAs were assembled into 262 clusters based on their primary sequence similarities. The putative translated proteins were classified into categories based on their function in accordance with significant similarity using the BlastX at NCBI FTP site and Pfam (Bateman et al. 2000) and SMART (Schultz et al. 2000) databases. The characterization of transcripts expressed in the fat body of Ae. aegypti at 24 hours post blood meal provides a basic tool for understanding the processes occurring in this organ and could identify putative new genes whose promoters can be used to specifically express transgenes in the fat bodies of Ae. aegypti.

## Introduction

Insect vectors are responsible for transmitting human pathogens such as dengue viruses and malaria parasites that account for millions of disease cases every year with associated high mortality and morbidity (TDR, 2004). While research devoted to alleviate the burden of these diseases on human populations focuses on the development of vaccines, preventive and therapeutic drugs, pioneering avenues for the control of diseases may also be opened from the studies of vector biology. The Anopheles gambiae genome project has provided the scientific community with a powerful tool for the analyses of gene expression in this malaria vector species (Holt et al. 2002). The 280-Mb An. gambiae genome and the tens of thousands of ESTs generated from RNA extracted from specific tissues such as midgut, fat body, salivary gland, hemocytes and antennae have made it possible to better understand mosquito biology and its interactions with pathogenic organisms. As expected, taking advantage of all the sequence information, and gene search and annotation, new types of experiments are possible that likely will result in novel observations and discoveries (Marinotti et al. 2005; Tabachnick 2003; Toure et al. 2004).

The genome of the mosquito, Aedes aegypti, the major vector of dengue viruses, is currently being sequenced (Severson et al. 2004), and once completed it will be a fundamental tool for studies on vector biology. The characterization of the trancriptomes of mosquitoes at various physiological stages as well as gene expression patterns in isolated tissues are fundamental additions to the complete genome description. The focus here is on the description of the transcriptome of Ae. aegypti vitellogenic fat bodies, which are dynamic tissues that participate in multiple biochemical functions of intermediate metabolism, including protein, amino acid, lipid and carbohydrate synthesis and storage, xenobiotic detoxification, and immune response (Bartholomay et al. 2004). Because dengue fever is the most prevalent mosquito-borne viral disease among human populations, causing 50 million infections, 500,000 cases of dengue hemorrhagic fever and least 12,000 deaths per year (Gubler 2002), the transcriptome of Ae. aegypti could prove useful in control of dengue.

A total of 800 randomly-picked cDNAs were sequenced and after quality validation, 589 cDNAs were assembled in 262 clusters, annotated and assigned gene ontology terminology.

## Materials and Methods

### Animals

Aedes aegypti (Rockefeller strain) were reared in a local facility at the Institute of Biomedical Sciences, University of São Paulo, Brazil. Temperature was maintained at 26°C, humidity at 80% and a 12/12 photoperiod. Larvae were fed on powdered rat food. Adult mosquitoes were given continuous access to a 10% sucrose solution and five-day old females were fed on anesthetized mice when required.

### Fat body cDNA library construction

Fat body of adult female mosquitoes, not including the body-wall integument, were dissected at 24 hrs post blood meal (PBM). Adult females were anesthetized on ice and dissected with a stereoscopic microscope in 0.15 M sodium chloride. Fat bodies were transferred to 500 μl of the TRIZOL reagent (Invitrogen) and mRNA extracted using the Micro-FastTrack mRNA isolation kit (Invitrogen). A PCR-based cDNA library was made following the instructions for the SMART cDNA library construction kit (Clontech). Four hundred nanograms of fat body mRNA were reverse-transcribed to cDNA using Superscript II RNase H-reverse transcriptase (Invitrogen) and the CDS III/3′ PCR primer (Clontech) for 1 h at 42°C. Second-strand synthesis was performed through a PCR-based protocol using the SMART III primer (Clontech) as the sense primer and the CDS III/3′ primer as antisense primer. These two primers create *Sfi* IA and B sites at the ends of nascent cDNA. Double-strand cDNA synthesis was carried out on a MJ Research Thermal cycler using the Platinum *Pfx* DNA polymerase. Amplification conditions were the following: 94°C for 2 min; 19 cycles of 94°C for 10 s and 68°C for 6 min. Double-stranded cDNA was treated immediately with proteinase K (0.8 μg/μl) for 20 min at 45°C. The double-stranded cDNA was digested with *Sfi* I for 2 h at 50°C. The cDNA then was fractionated using columns provide by the manufacturer (Clontech). Fractions containing cDNA of more than 400 base pairs (bp) in length were pooled and concentrated to a volume of 7 μl. The concentrated cDNA was ligated into a Lambda TriplEx2 vector (Clontech), and the resulting ligation reaction packaged using the Gigapack Gold III from Stratagene/Biocrest. The library was plated by infecting log-phase XL1-Blue cells (Clontech).

### Sequencing and analysis of the Ae. aegypti cDNA library

Randomly-picked cDNA clones from the cDNA library were sequenced, assembled and analyzed (Durham *et al.* 2005; Calvo et al. 2004), using the CAP 3 program (Huang and Madan 1999). BLASTX searches were done locally from programs obtained at the NCBI FTP site (ftp://ftp.ncbi.nih.gov/blast/executables/) (Altschul et al. 1997). All the ESTs were deposited in dbEST at NCBI. Accession numbers for sequences originating from the Ae. aegypti cDNA library are given as DT366744 to DT367332 corresponds to the referenced gene product.

## Results and Discussion

### Organization of the transcriptome information

A total of 589 cDNA inserts were assembled into 262 clusters. Thirty-one clusters (237 cDNAs) were identified as corresponding to nuclear or mitochondrial rRNAs and were not investigated further. The 351 remaining cDNAs were grouped in 231 clusters and classified in 21 categories, according to function or putative assigned functions (Tables 1 and 2, and a supplementary table where cDNA is classified in categories: http://lineu.icb.usp.br/~mcapurro/aafatbody.zip).

**Table 1.**
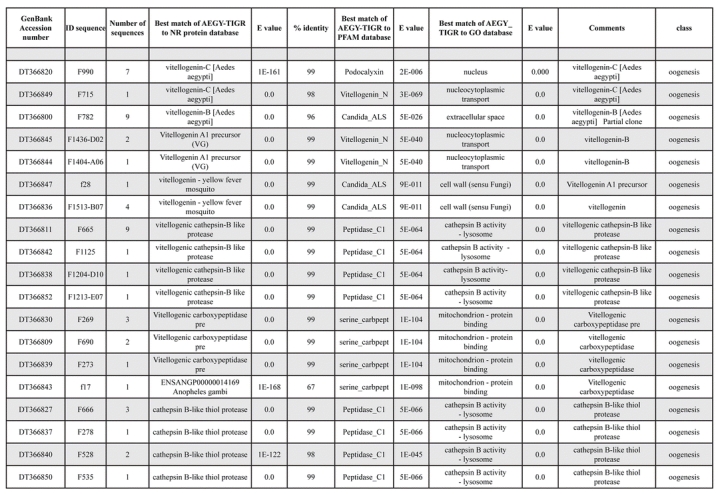
Aedes aegypti vitelogenic fat body cDNA cluster encoding proteins

**Table 1.**
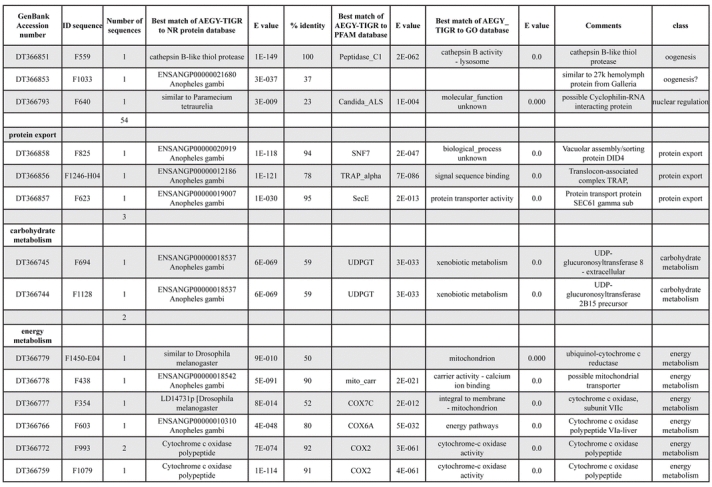
Continued

**Table 1.**
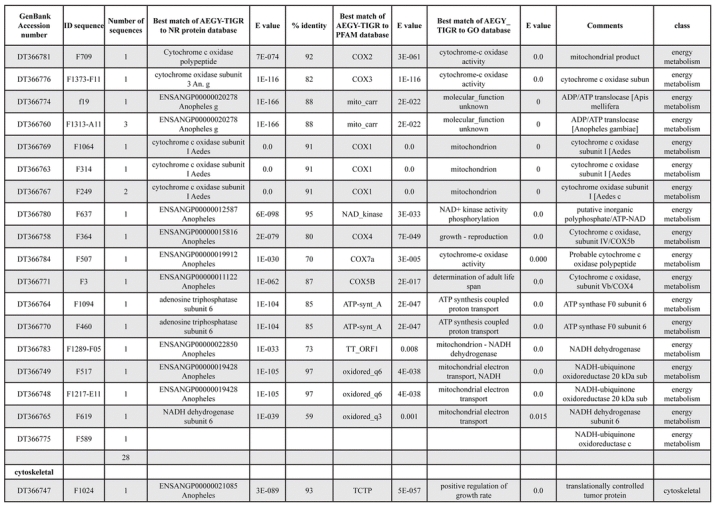
Continued

**Table 1.**
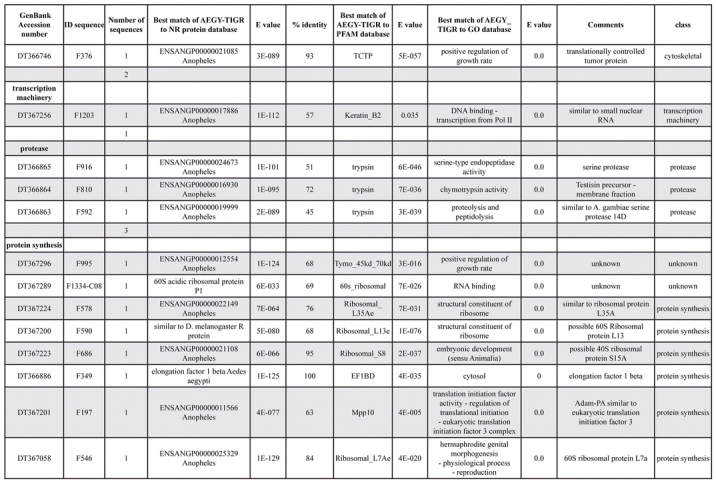
Continued

**Table 1.**
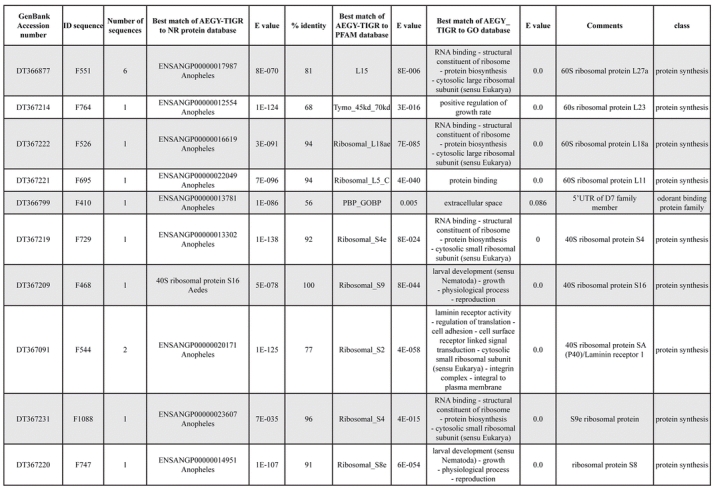
Continued

**Table 1.**
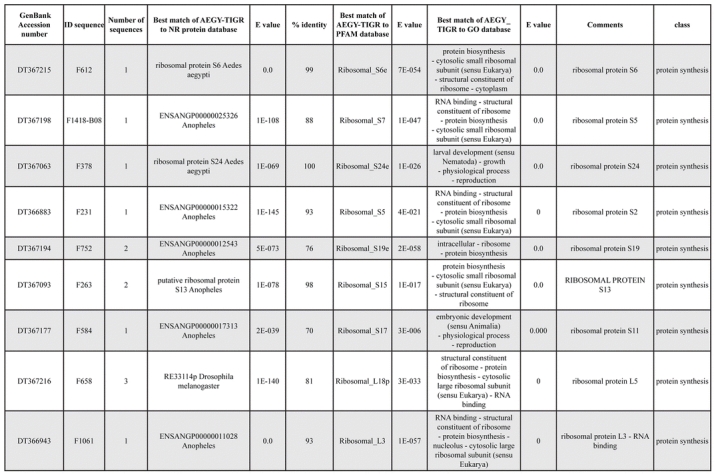
Continued

**Table 1.**
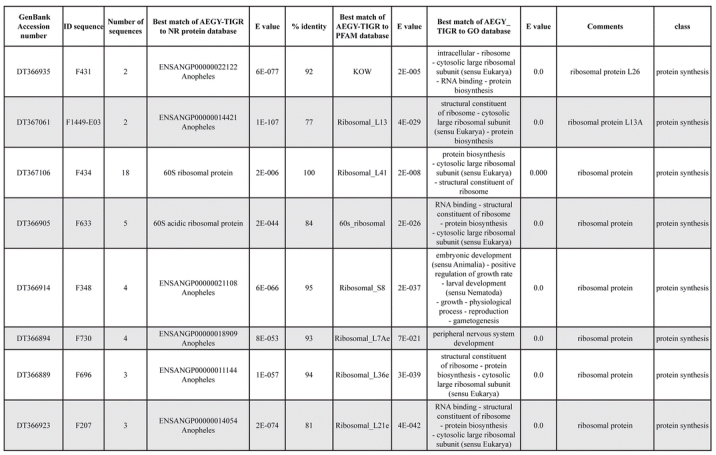
Continued

**Table 1.**
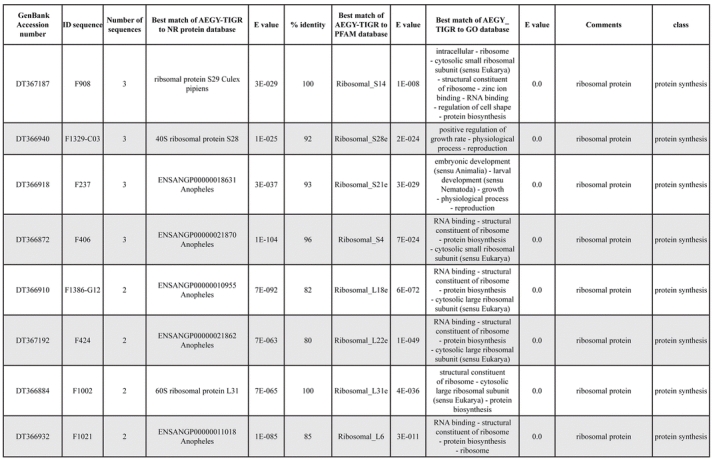
Continued

**Table 1.**
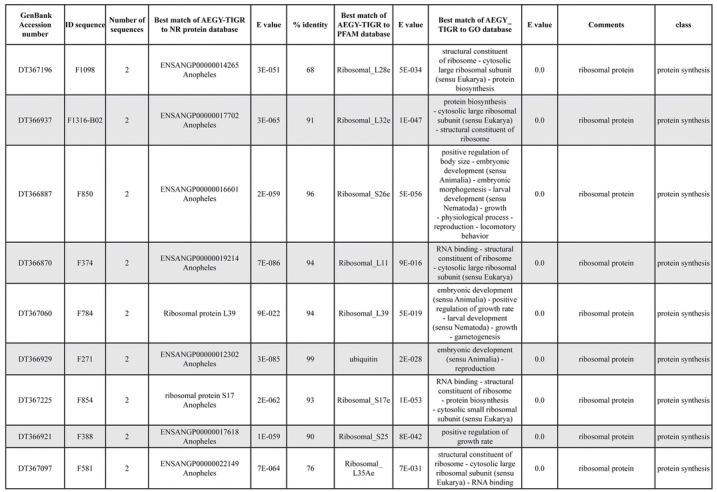
Continued

**Table 1.**
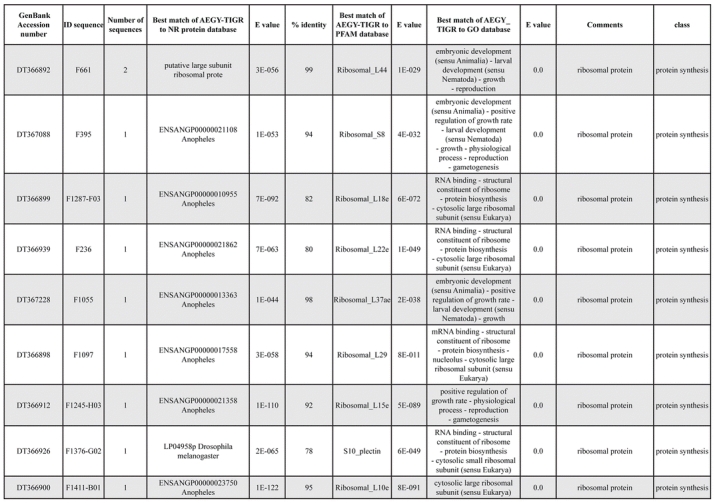
Continued

**Table 1.**
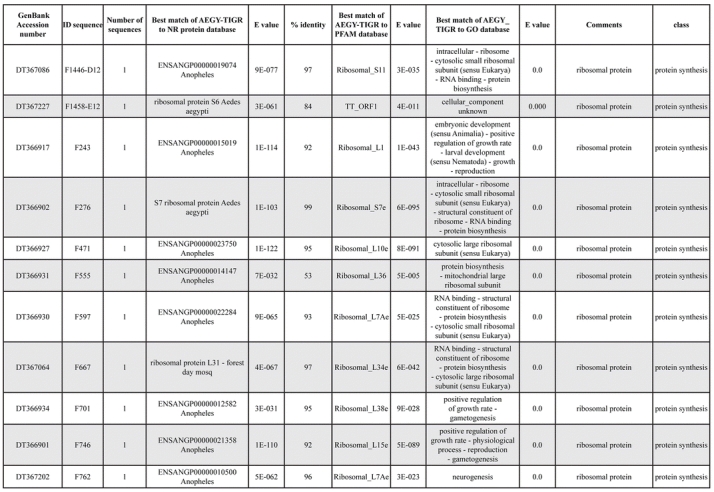
Continued

**Table 1.**
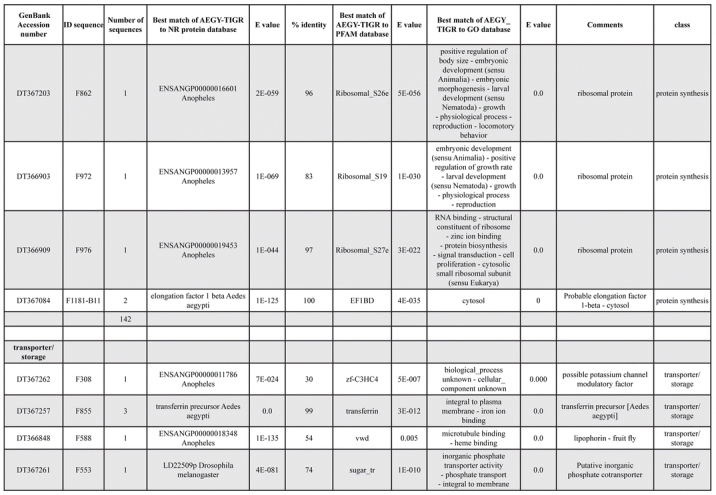
Continued

**Table 1.**
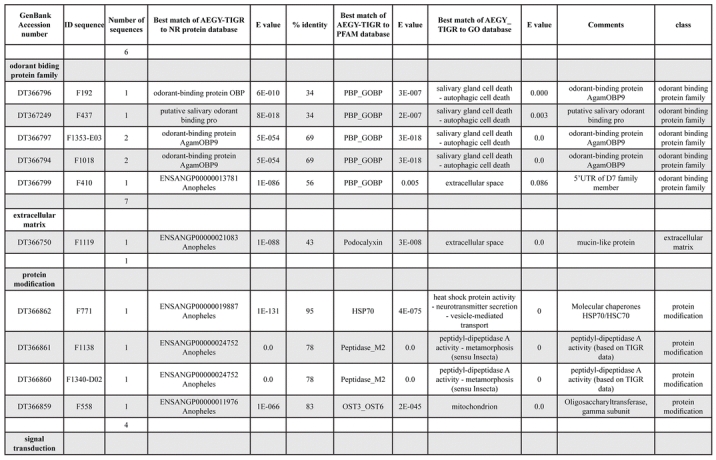
Continued

**Table 1.**
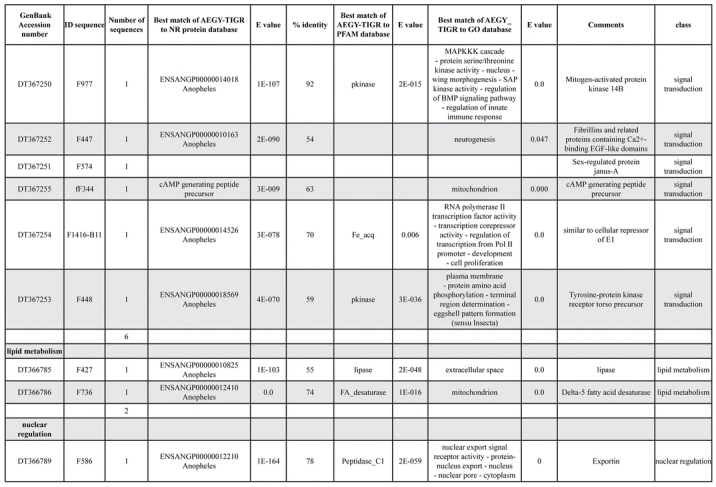
Continued

**Table 1.**
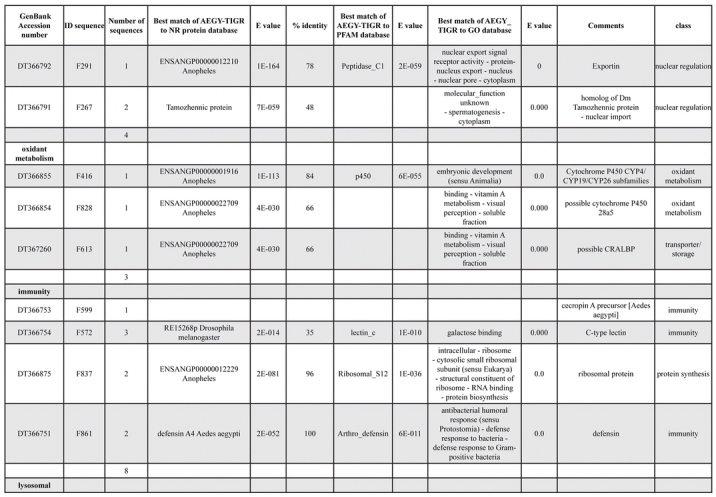
Continued

**Table 1.**
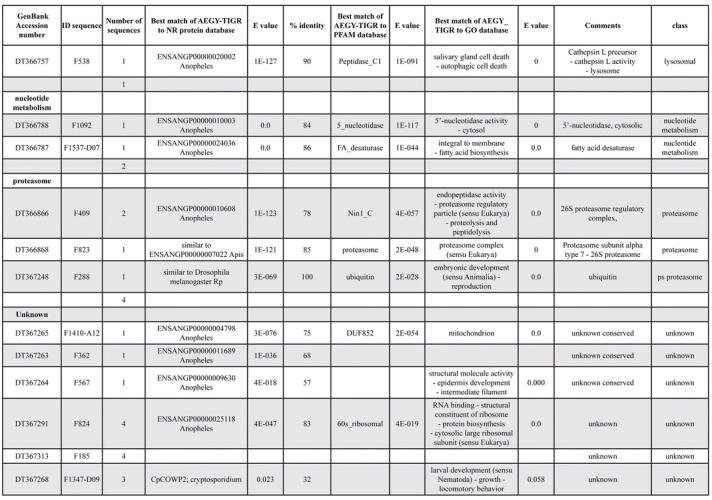
Continued

**Table 1.**
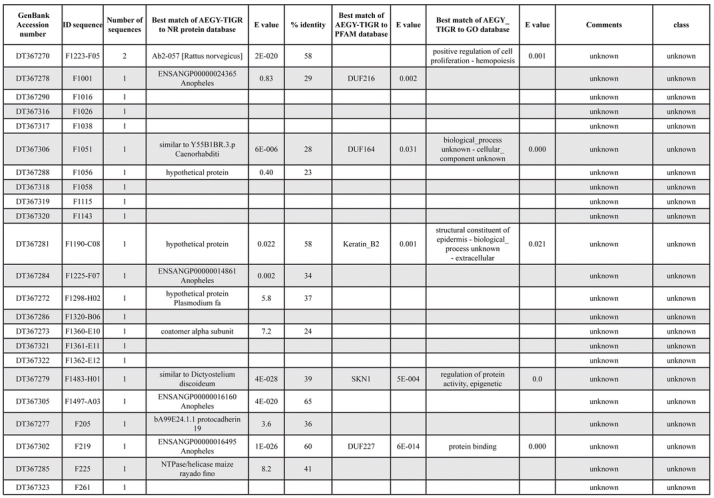
Continued

**Table 1.**
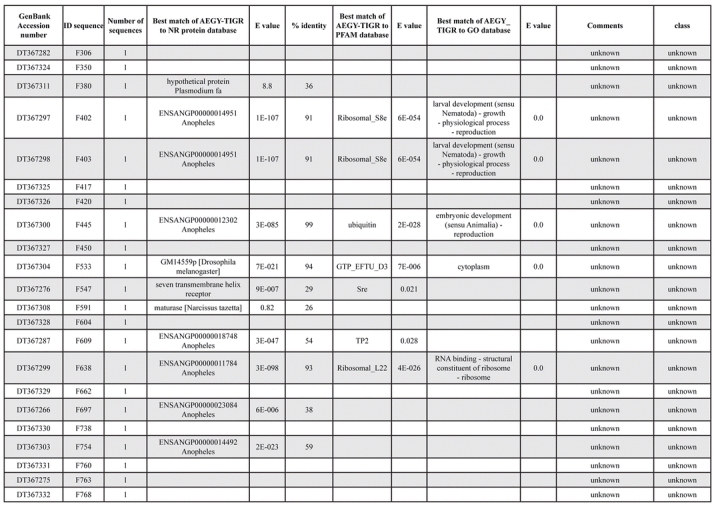
Continued

**Table 1.**
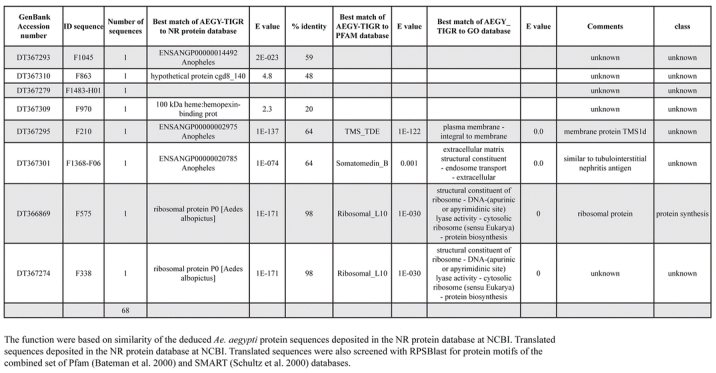
Continued

**Table 2.**
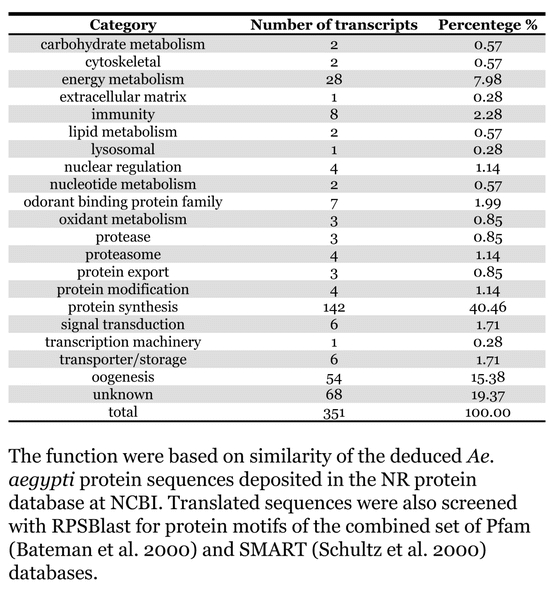
Division of the transcriptis according to their functions

The known or putative functional assignments were based on similarity of the deduced protein sequences to previously-described molecules deposited in the NR protein database at NCBI. Translated sequences also were screened with RPSBlast for protein motifs of the combined set of Pfam (Bateman et al. 2000) and SMART (Schultz et al. 2000) databases (also known as the Conserved Domains Database [CDD]) (Table 1). The sequences were compared to those available through the Ae. aegypti genome-sequencing project, at the TIGR web site (09/22/2004).

The vast majority of identified ESTs were represented only once in the database, indicating the high complexity of the fat body tissue. Fifty-seven contigs correspond to cDNAs that occur more than once in the database, may be indicative of a higher level of gene expression. Transcripts encoding proteins of the unknown group (68 ESTs), which did not show significant similarity with known proteins, could represent novel proteins unique to Ae. aegypti fat bodies and require further investigation.

### Description of the transcriptome

**Figure 1.**
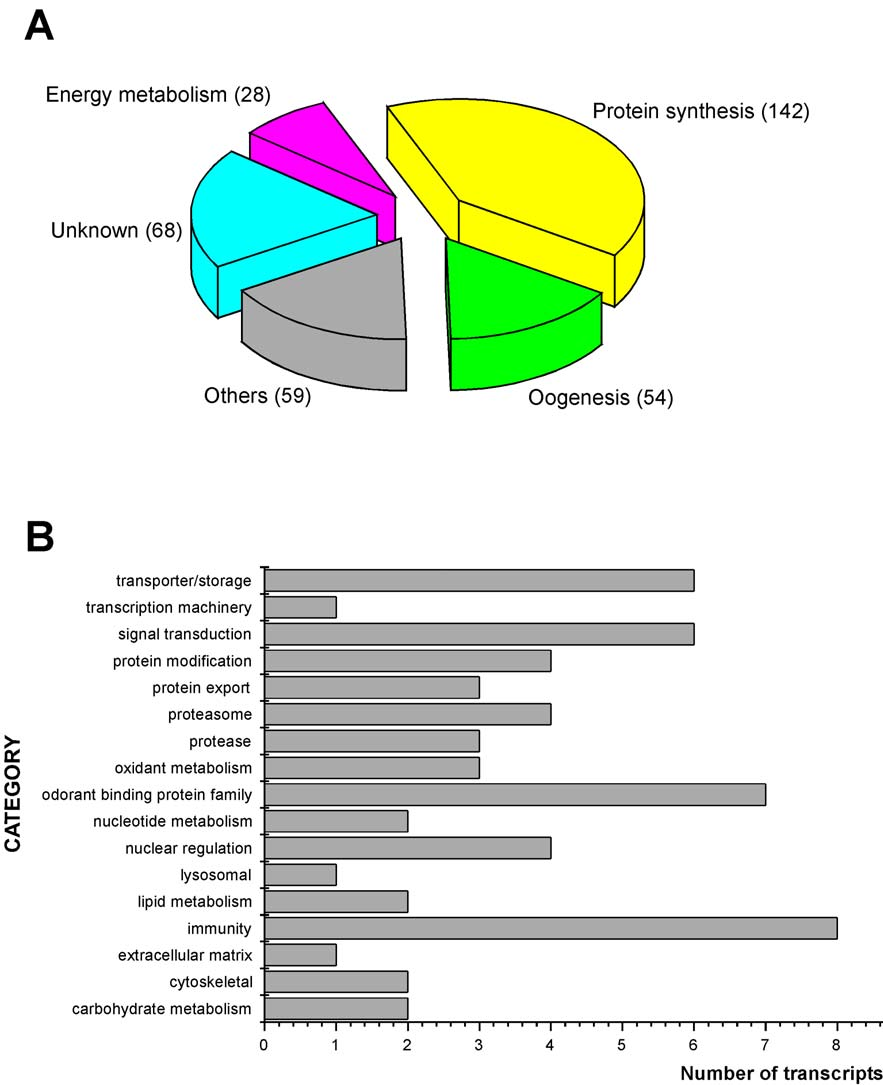
Number of sequences of the Aedes aegypti fat body cDNA library. The transcript categories are more represented (A) and less represented (B - Others). The sequences were classified in accordance with function based on similarity of the deduced Ae. aegypti protein sequences deposited in the NR protein database at NCBI. Translated sequences were also screened with RPSBlast for protein motifs of the combined set of Pfam (Bateman et al. 2000) and SMART (Schultz et al 2000) databases. See also Table 2.

All of the cDNAs reported here were divided into 21 groups according to their predicted function by gene ontology (Ashburner et al. 2000), (Figure 1 and Table 2). In total, 142 sequences distributed in 75 clusters, representing 40.46% of the total of fat body ESTs database, correspond to genes whose products are associated with protein synthesis. This is consistent with the synthetic capability of the fat bodies. The majority of these cDNAs encode ribosomal proteins (67 clusters). The presence of transcripts encoding an elongation factor and a translation initiation factor supports further the conclusion of an abundant protein synthetic activity of this organ.

Six transcripts encoding molecules associated with post-translational modification of proteins and protein transport pathways were found. Furthermore, transcripts of mitochondrial components are among the most represented in the database and this is consistent with the energy requirements for protein synthesis during vitellogenesis. Sequences related to energy metabolism (28 sequences distributed in 24 clusters) correspond largely to cytochrome c oxidase, ATP synthase and NADH dehydrogenase. Two transcripts involved in oxidative metabolism were identified as members of the cytochrome P450 family.

Transcripts involved with a wide variety of functions such as carbohydrate, lipid and nucleotide metabolism, transport, storage, signal transduction, lysosomal and proteosome activities and, nuclear regulation, were found among the sequenced cDNAs. All of these were represented only once or twice in the database, except for a transferrin and an odorant binding protein-like transcript, which were represented three and four times, respectively. The function of Ae. aegypti transferrin has not been determined, but these molecules may be involved in iron transport, oogenesis and innate immunity against parasites and pathogens (Harizanova et al. 2005). Insect odorant binding proteins and pheromone binding proteins (Hekmat-Scafe et al. 2000) are expressed mainly in the insect antennae and they are proposed to bind small hydrophobic odorant molecules, carry and present them to the olfactory receptors. Proteins structurally related to odorant binding proteins have been identified in non-sensory organs and in the hemolymph of insects, where they might carry hydrophobic ligands related to a variety of functions (Biessmann et al. 2002; Calvo et al. 2004). A hemolymph protein with sequence and structural characteristics similar to odorant binding proteins, designated THP12, was described in Tenebrio molitor as a carrier protein, associated with the transport of small hydrophobic ligands through the hemolymph (Graham et al. 2001; Rothemund et al. 1999). These Ae. aegypti cDNAs may correspond to novel mosquito proteins yet to be described.

Three immunity-related transcripts were found, corresponding to molecules with high amino acid similarity (93%) to the C-type lectin found in Drosophila melanogaster (Haq et al. 1996), defensin and cecropin of Ae. aegypti. One sequence was identified as a cecropin precursor, also from Ae. aegypti. The cecropins have antibacterial and antifungal activity (Zheng and Zheng, 2002) while defensins are antimicrobial peptides that are activated in the presence of Gram-positive or negative bacteria. These peptides also are activated by infection with filarial worms (Bartholomay et al. 2004).

Transcripts related to vitellogenesis (Table 1 and Table 2), represent 15.38% of the fat body transcriptome (54 sequences, 22 clusters) at 24 h PBM, and encode vitellogenins, vitellogenic cathepsin-B and vitellogenic carboxypeptidase. These data are in agreement with the previously-described increase of vitellogenic proteins expression in mosquitoes following a blood meal (Kokoza et al., 2001). Transcripts representing three distinct vitellogenins, three cathepsin Bs and two vitellogenic carboxypeptidases were observed in the analyzed set of cDNAs. Multiple vitellogenin genes in the genome of Ae. aegypti have been described (Hamblin et al. 1987) while the multiple transcripts with similarity to cathepsin B and vitellogenic carboxypeptidase indicate that these proteins may be encoded by more than one gene as well. It has been already established that vitellogenin, vitellogenic carboxypeptidase and cathepsin B are synthesized by the fat bodies in response to a blood meal, secreted into the hemolymph and accumulate in ovaries, where they are deposited in the oocytes during development (Raikhel, 1987; Raikhel, et al., 2002; Raikhel and Lea, 1983; Ribeiro, 2003).

### Final remarks

The sequencing of 800 Ae. aegypti fat body-derived cDNAs, and their annotation and organization provides a general picture of the metabolic state of the tissue at 24 h PBM. The insect fat body is a complex tissue where many indispensable processes of intermediate metabolism, storage and immunity occur, however its major function is synthesis and export of proteins (ex. hexamerins, transport proteins and vitellogenic proteins, etc).

This survey revealed strongly-expressed genes in the fat bodies of Ae. aegypti and is useful for the purpose of identifying genes whose promoters can be used for driving the expression of anti-pathogen molecules in transgenic mosquitoes, (James et al. 1999). In addition, the use of transgenic mosquito strains in which a female-specific promoter controls the expression of a lethal gene has been proposed as a powerful technique to generate a male-only population useful for a variation of the Sterile Insect Release technique (Thomas et al. 2000). This requires that a strain of the target organism carry a dominant, sex-specific lethal gene whose expression can be repressed in the laboratory or insectary under controlled conditions. Some of these genes may be suitable targets for new insecticides or strategies to block parasite development within the vector. Other advances could result from the comparative analysis of regulatory *cis*-acting elements found in the promoters of selected genes. For example, searches for conserved motifs may identify common regulatory motifs in the promoters of genes strongly-expressed in the fat bodies of mosquitoes. It is anticipated that genes expressed exclusively in the fat body, mostly those regulated coordinately, may share transcription factor-binding sites in their promoters and other regulatory sequences in the respective UTRs.
